# Primary Suture of War Wounds

**Published:** 1918-03

**Authors:** 


### SURGICAL

**Primary Suture of War Wounds.** By George Gross, H. Tissier, L. Boudard, F. Di Chiara, and L. Grimault. Trans. and Abs. from the *Bulletin et Mémoires de la Société de Chirurgie de Paris,* Oct. 16, 1917.

The authors write in confirmation of the statement made earlier by Tissier that *war wounds which are not infected by the streptococcus may be sutured primarily, and, that if they receive the proper surgical treatment, they ought to heal by first intention* [1].

1. *Annales de l'Institut Pasteur*, Dec., 1916; *Bull. de l'Acad. de Méd.*, Oct., 1916; *Arch. de Méd. Milit.*, Dec., 1916; *Bull. et Mém. de la Soc. de Chir.*, July 10, 1917. (For an abstract of the last see *The Medical Bulletin* for Nov. 1917, p. 33.)

The authors, on the basis of broader experience, declare that *primary suture is at present the rational treatment for war wounds.*

Out of a total of 549 cases operated in the authors' Ambulance, 430 were treated by primary suture. This fact is especially significant because the cases treated were of a severe type, 209 of them were fractures. Out of 759 sutures actually performed, the results were 675 reunions by first intention, 47 partial disunions, and 37 deliberate disunions.

The authors describe the evolution of the various types of cases treated by primary suture :

*Normal Progress.* — The vast majority of cases belong to this class. 675 out of a total of 759 of the sutures performed in the Ambulance could be regarded as normal. Such cases are marked by an absence of pain. After the second day the patient does not complain of suffering. Patients treated by secondary suture and those with unsutured wounds suffer continuously.

The general health is almost perfect. If the wound is in an upper limb, the patient can begin to get up after the second day. All the usual indications of good health are to be observed and the temperature is only slightly raised, rarely beyond 38° C. for four or five days.

The dressing applied at the time of the operation need not be removed before the tenth day. The stitches may then be removed leaving a perfectly normal scar. Patients may be discharged as healed within from 15 to 20 days after the operation.

Such a course of healing is by no means limited to light wounds. It is possible with many of the most serious types of wounds, and especially fractures.

*Sutures Attended with Complications.* — In a very small percentage of the cases slight complications may occur. In spite of these, union by first intention may often be obtained, or a union with a very limited superficial infection. The temperature gives timely warning. It may remain at from 38° to 39° C. for 8 or 10 days. About the fifth or sixth day it may show a slight increase, but it falls again gradually. The general health is good and the pulse strong; the latter is never feeble or rapid as in cases of serious infection.

The infection shows itself in a reddish tint observed around one or two of the stitches, and there may be a slight local swelling and small purulent eruptions about the wound. When the infection is of this mild type, the healing is very little delayed.

There is a more serious type of infection, however, which sets in with a high temperature the very day of the operation. The temperature may remain at from 39° to 40° for two or three days, and then fall more or less abruptly.

In such cases it generally suffices to insert a pair of Kocher forceps between two of the stitches, thus allowing the escape of any hematoma or gas bubbles that may have formed. The healing is not likely to be delayed unless stitches are removed.

In a very few cases (the authors have observed only 9) the skin may appear tense and discolored, with a cherry tint, to a considerable distance. Gas may be noticed under the suture and the muscle may be tympanitic. At the start, the swelling has more of a cherry tint than is the case in gas gangrene. There is, however, no serious rise in temperature, and the general condition is excellent. Such cases must be controlled bacteriologically. It is not necessary to open the suture, if it is clearly ascertained that the streptococcus is not present. In a few cases there may be suppuration and gaseous abcesses may have to be opened.

*Streptococcic Infection.* — As soon as it is clearly ascertained that the streptococcus is present, the suture should be opened. Often these cases show very few signs of infection until the stitches are removed. The wound may begin to open spontaneously. It is best to open the wound at once.

*Gaseous Gangrene.* — A mild form of gaseous gangrene is sometimes noticed at the end of a few hours. The patient complains constantly and his general condition is bad. His pulse is rapid and his tongue dry. The skin in the region of the suture has the tint of wine dregs and is mottled. Gas may escape from the wound. If the wound is immediately opened and properly cleansed this condition may be checked before it becomes dangerous. It rarely leads to the spreading type of gas gangrene.

A sub-acute but highly toxemic form of gaseous gangrene may sometimes set in, producing death almost without gangrene. Such cases are very rare, and they ought not to be attributed to the primary suture. They occur also when wounds are treated surgically without suture. Furthermore, the wound may be at once reopened for surgical cleansing.

Out of all the cases treated, there were only 23 deaths. All but three of these were due to clearly unavoidable causes (visceral wounds, shock, etc.). Only three cases of flesh wounds treated by suture were fatal. These cases were typical of badly infected wounds, and the authors believe that no form of surgical intervention could have saved them.

*Bacteriology.* — Infection with the streptococcus is mainly to be feared. The authors recommend that all such cases be isolated and specially treated.

They state that bacteriological tests for this organism may be completed according to Tissier's technique within six hours. Direct examinations of pus and serosity are valueless. Culture tests alone are reliable. The streptococcus grows quickly only in liquid media. Characteristic colonies in long twisted chains may be thus obtained at a temperature of 37° C. Other species are recognizable later in solid media. Anaerobes can at present be recognized only by their morphological characteristics and by their cultures. Numerous specimens from all parts of the wound should be examined. The technique of the test is described as follows:

" Ordinary broth, lactose agar, and Veillon agar, should be inoculated. Cultures should be taken, if possible, between the 14th and 18th hours after the wound. Earlier there is danger of collecting an incomplete flora; later, during the course of the second day, when the putrefaction has set in, there is danger of procuring only anaerobes. It often appears that the B. perfringens is then the only organism. If this condition exists, it is necessary to wait until the 4th day in order to determine the initial flora. Since it is highly important that the results of the bacteriological tests hould be known the second day, especially in difficult cases, cultures should be taken at the earliest opportunity. One should not hesitate to repeat the findings and the sowings. Even during the second and third days, these may give valuable information as to the intensity of the putrefaction. As late as the fifth and sixth days they may give information as to the nature and progress of gaseous abscesses. "

*Surgical Technique.* — The first step in cleansing a wound should be the complete excision of the entrance orifice, and next, if the wound is of the seton type, of the exit orifice. An elliptical incision should be made large enough to permit of the complete excision of the deeper parts of the missile tract. The authors consider any attempt to explore the tract to be either useless or dangerous. Its course may be determined radioscopically. There ought to be no blind surgical action of any sort. Cleansing of a wound is a dangerous and misleading term. Only a generous excision of soiled or contused tissues should be practised. It is always clear when healthy tissue with a normal blood supply has been reached.

Three types of operation may be performed :

1. Laying open of the seton or missile tract under the skin, and its complete removal by a single operation. This may be done if the tract is not too deep.

2. The excision of the wounded tissue *en masse*. This is the desirable method in the case of wide wounds, especially if they are not deep.

3. Most frequently a cone-shaped excision is employed. If the missile is in the wound, the apex of the cone should extend beyond the missile or any fracture caused by it. This operation is performed most minutely with scissors. It is highly important that every particle of contused tissue be removed. In the case of a deep seton wound a double cone operation is imperative. The two apices should join.

When there are bony lesions, the authors recommend a conservative treatment of splinters. They recommend the removal of only the detached fragments. They believe that adherent splinters should be respected, or at most regularized. Bone cavities in which missiles are lodged should, if possible, be leveled. The bone cavity should be treated with ether and iodoform. Houdard has had four excellent results from fatty grafts taken from the buttocks.

In joint wounds the authors, whenever possible, practise the Loubat operation, with primary resection of the more extensive wounds.

As an antiseptic they use only ether, which, in the case of bone operations, they combine with iodoform.

The *suture* itself requires perfect hemostasis and the perfect union of the faces of the wound. The authors believe that catgut is likely to lead to infection, and they prefer bronze wire. They think that the stitches should not be removed until the 10th or 12th day.

The suture may be performed in all cases in which the bacteriologist does not discover the streptococcus. Out of a total of 727 wounded, the authors refrained from performing sutures for other reasons in only 119 cases. These were chiefly cases of multiple wounds, severe shock, extensive, old, or badly infected wounds, wounds with imbedded projectiles, wounds involving serious vessel lesions, wounds involving a large loss of skin, and wounds that defied suture.

